# Supplementary material for: Association of early viral lower respiratory infections and subsequent development of atopy, a systematic review and meta-analysis of cohort studies
Source: PLoS One. 2020 Apr 24;15(4):e0231816. doi: 10.1371/journal.pone.0231816 (PMC7182231; doi:10.1371/journal.pone.0231816)
Supplement: S2 Table — (PDF) [file pone.0231816.s002.pdf]

1.2. Supplementary Table 2. Items for risk of bias assessment

**Newcastle – Ottawa Scale for case cohort studies**	**One star (1)**	**No star (0)**
**Selection**		
1) Representativeness of LRTI cohort	Representative of the population source	Specific population or no description of the population source
2) Selection of patients without LRTI cohort	From the same population source	From a different population source or no description of the population source
3) Ascertainment of LRTI exposition	From secure record or structured interview	Self report or no description
4) Demonstration that atopy was not present at the beginning of the study	Yes	No
**Comparability**		
1) Comparability of infants with and without LRTI in infancy age at inclusion	Yes	No
2) Comparability of infants with and without LRTI in infancy for a second important factor	Yes	No
**Outcome**		
1) Assessment of the atopy	Skin prick and serum tests or record linkage	Self report or no description
2) Was follow-up long enough for the development of atopy	Yes	No
3) Complete follow up of all subjects accounted for 80%	Yes	No or no description
Total score	**9**	**0**
**Interpretation of the two risk of bias tools**<br><br>• 6-9: Low risk of bias<br>• 0-5: High risk of bias		
