# Supplementary material for: Association of early viral lower respiratory infections and subsequent development of atopy, a systematic review and meta-analysis of cohort studies
Source: PLoS One. 2020 Apr 24;15(4):e0231816. doi: 10.1371/journal.pone.0231816 (PMC7182231; doi:10.1371/journal.pone.0231816)
Supplement: S3 Table — (PDF) [file pone.0231816.s003.pdf]

1.3. Supplementary table 3. Individual characteristics of included studies

Author, Date	Period of LRTI	Timing_case recrutement	follow-up delay (Years)_case	Age_cases at recrutment	Age_cases at interview	Type of LRTI	LRTI case definition	First episode of LRTI	Country	WHO_Region	Sampling method	Case Hospitalisation	Male_cases	Male_controls	Atopy detection test	viruses_screened	sample types	Detection assays
Fjærli, 2005	1993-1994	Prospective	7 years	5.1 months	7 years	Bronchiolitis	Infants presenting with respiratory insufficiency such as tachypnoe, intercostal retractions, increased mucus production and soar coughing were eligible for follow-up	Unclear/Not reported	Norway	Europe	Consecutive	Yes	57,1	56,25	Unclear/Not reported	HRSV	Nasopharyngeal aspirate	EIA. Immunofluorescence
García-García, 2007	Oct/2000-Jun/2005	Prospective	Unclear/Not reported	122 ± 133 days	4.03 years	Bronchiolitis	All the classic criteria, present in an initial episode of acute onset expiratory dyspnea with previous signs of viral respiratory infection (whether or not this was associated to respiratory distress or pneumonia) were applied in diagnosing bronchiolitis.	yes	Spain	Europe	Consecutive	Yes	53,1	43,3	Unclear/Not reported	HRSV	Unclear/Not reported	Immunofluorescence. RT-PCR
García-García, 2007	Oct/2000-Jun/2005	Prospective	Unclear/Not reported	140 ± 130 days	3.7 years	Bronchiolitis	All the classic criteria, present in an initial episode of acute onset expiratory dyspnea with previous signs of viral respiratory infection (whether or not this was associated to respiratory distress or pneumonia) were applied in diagnosing bronchiolitis.	yes	Spain	Europe	Consecutive	Yes	60,8	43,3	Unclear/Not reported	HMPV	Unclear/Not reported	RT-PCR
Henderson, 2005	Apr/1991-Dec-1992	Retrospective	3-7 years	12 months	30-42 months	Bronchiolitis	Hospital discharge diagnostic code	Yes	United Kingdom	Europe	Consecutive	Yes	Unclear/Not reported	Unclear/Not reported	Skin prick test	HRSV	Nasopharyngeal aspirate	Immunofluorescence. Culture
Juntti, 2003	1991-1994	Prospective	6-10 years	4.0 months	6-10 years	LRTI + bronchiolitis	Hospitalized with an HRSV infection	Unclear/Not reported	Finland	Europe	Consecutive	Yes	61,8	Unclear/Not reported	skin prick and Serum tests	HRSV	Nasopharyngeal secretions	Antibody assays
Korppi, 2004	1981-1982	Prospective	18-19 years	< 2 years	18 à 20 years	Bronchiolitis + Pneumonia	Hospitalized with bronchiolitis or pneumonia	Unclear/Not reported	Finland	Europe	Consecutive	yes	Unclear/Not reported	Unclear/Not reported	Skin prick test	HRSV	Unclear/Not reported	Unclear/Not reported
Mikalsen, 2012	1997-1998	Prospective	11 years	3 months	11.3 years	Bronchiolitis	Bronchiolitis was defined clinically as an acute febrile respiratory illness with tachypnoea, dyspnoea, prolonged expiration and wheeze on auscultation of the chest.	Unclear/Not reported	Norway	Europe	Consecutive	Yes	61,2	60,2	Skin prick test	HRSV	Nasopharyngeal mucus	Immunofluorescence directe
Murray, 1992	1979-1981	Prospective	5.5 years	103 days	65-78 months	Bronchiolitis	The criterion for entry was the clinical diagnosis of acute bronchiolitis in a child with no history of a previous similar illness. All had cough, tachypnoea, and feeding difficulties associated with hyperinflation, recession, fine crepitations, and often rhonchi.	Yes	United Kingdom	Europe	Consecutive	Yes	49,3	Unclear/Not reported	Skin prick test	HRSV	Unclear/Not reported	Immunofluorescence. Culture
Nicolai, 2017	Oct/2009-May/2014	Retrospective	12. 24 and 36 months	2.9 months	Unclear/Not reported	LRTI + bronchiolitis	For cases, on admission, we searched the clinical records for data on the following clinical and laboratory variables: presence of rhinitis, pharyngitis, otitis, vomiting, diarrhea and fever (temperature, >37.5°C).	Unclear/Not reported	Italy	Europe	Consecutive	Yes	61,2	58,4	Unclear/Not reported	HRSV. Influenza. HCoV. HAdV. RV. HPIV. HMPV. HBoV	Nasopharyngeal swabs	RT-PCR
Peña Zarza, 2012	Oct/2006-Apr/2007	Prospective	2 years	3.24 months	Unclear/Not reported	Bronchiolitis	The acute phase of bronchiolitis (phase 1) were related with wheezing episodes diagnosed by a clinician in the 2 years following the first one.	Yes	Spain	Europe	Consecutive	Unclear/Not reported	71,4	50	Unclear/Not reported	HRSV	Nasopharyngeal secretions	Immunofluorescence et par la Culture
Poorisrisak, 2010	Jan/1994-Dec/2003	Retrospective	Unclear/Not reported	10.6 months	7.6 years	LRTI + bronchiolitis	The Danish National Patient Registry records all hospitalizations for HRSV infection based on the International Statistical Classifi cation of Diseases and Related Health Problems, 10th revision.	Unclear/Not reported	Denmark	Europe	Consecutive	Yes	Unclear/Not reported	Unclear/Not reported	Skin prick test	HRSV	Unclear/Not reported	EIA. Immunofluorescence
Poulsen, 2006	1994-1997	Retrospective	Unclear/Not reported	0.86 ± 0.86 year	6.80 years	LRTI + bronchiolitis	This birth cohort was followed up by weekly visits to detect possible RSV ALRI cases according to WHO criteria.18	Unclear/Not reported	Guinea-Bissau	Africa	Consecutive	Yes/No	Unclear/Not reported	Unclear/Not reported	skin prick and Serum tests	HRSV	Nasopharyngeal aspirate	ELISA
Pullan, 1982	Unclear/Not reported	Retrospective	10 years	14 weeks	Unclear/Not reported	LRTI + bronchiolitis	Ten of the 18 children diagnosed as having bronchitis, 109 of the 144 diagnosed as having bronchiolitis, and 11 of the 18 diagnosed as having pneumonia during the original admission (definitions as suggested by Court11) were seen for assessment.	Yes	United Kingdom	Europe	Consecutive	Unclear/Not reported	Unclear/Not reported	Unclear/Not reported	Unclear/Not reported	HRSV	Unclear/Not reported	Unclear/Not reported
Ruotsalainen, 2010	1981-1982	Prospective	12 months	< 2 years	27.0 years (range 26.4 –28.5)	LRTI + bronchiolitis	The presentation of RSV LRI in infancy was classified by clinical and radiologic criteria: obstruction - infiltration + in 12, obstruction + infiltration - in 14, and obstruction + infiltration + in 14 cases.	Unclear/Not reported	Finland	Europe	Consecutive	yes	Unclear/Not reported	Unclear/Not reported	Unclear/Not reported	HRSV	Unclear/Not reported	Antigen and antibody assays
Ruotsalainen, 2013	1992-1993	Prospective	15.6 years	< 2 years	15 à 18 years	Bronchiolitis	The diagnostic criteria of bronchiolitis were the presence of respiratory infection and wheezing requiring treatment in hospital.	Unclear/Not reported	Finland	Europe	Consecutive	Yes	67,1	67	Serum test	Seven viruses including HRSV and RV	Nasopharyngeal aspirate	Antigen detection. Antibody assays. RT-PCR
Schauer, 2002	Dec/1999-Apr/2001	Prospective	Unclear/Not reported	16 weeks	0.7-1.5 years	Bronchiolitis	The clinical diagnosis of acute bronchiolitis required the presence of tachypnoea, wheeze, a prolonged expiratory phase, and crackles on auscultation, recorded at some time during the admission.	Unclear/Not reported	Germany	Europe	Consecutive	Yes	42,8	42,8	Serum test	HRSV	Nasopharyngeal aspirate	EIA
Sigurs, 1995	1989-1990	Prospective	1. 2 and 3 years	3.5 months	1 year	Bronchiolitis	Bronchiolitis was defined as tachypnea, prolonged expiration, dyspnea, and wheezing on auscultation of the chest after a brief prodrome of upper respiratory symptoms, as proposed by Ruuskanen and Ogra.1	Unclear/Not reported	Sweden	Europe	Consecutive	Yes	44,6	45,1	skin prick and Serum tests	HRSV	Nasopharyngeal secretions	EIA
Sigurs, 1995	1989-1990	Prospective	1. 2 and 3 years	3.5 months	3 years	Bronchiolitis	Bronchiolitis was defined as tachypnea, prolonged expiration, dyspnea, and wheezing on auscultation of the chest after a brief prodrome of upper respiratory symptoms, as proposed by Ruuskanen and Ogra.1	Unclear/Not reported	Sweden	Europe	Consecutive	Yes	Unclear/Not reported	Unclear/Not reported	skin prick and Serum tests	HRSV	Nasopharyngeal secretions	EIA
Sigurs, 2000	Dec/1989-Apr/1990	Prospective	6.5 years	116 days	7.12-8.08 years	Bronchiolitis	Bronchiolitis was defined as tachypnea, prolonged expiration, dyspnea, and wheezing on auscultation of the chest after a brief prodrome of upper respiratory symptoms, as proposed by Ruuskanen and Ogra.1	Unclear/Not reported	Sweden	Europe	Consecutive	Yes	44,6	45,1	skin prick and Serum tests	HRSV	Nasopharyngeal secretions	EIA
Sigurs, 2005	Dec/1989-Apr/1990	Prospective	12.5 years	116 days	13.0-14.0 years	Bronchiolitis	Bronchiolitis was defined as tachypnea, prolonged expiration, dyspnea, and wheezing on auscultation of the chest after a brief prodrome of upper respiratory symptoms, as proposed by Ruuskanen and Ogra.1	Unclear/Not reported	Sweden	Europe	Consecutive	Yes	43,5	44,6	skin prick and Serum tests	HRSV	Unclear/Not reported	Unclear/Not reported
Sigurs, 2010	Dec/1989-Apr/1990	Prospective	Unclear/Not reported	116 days	18 years	Bronchiolitis	Bronchiolitis was defined as tachypnea, prolonged expiration, dyspnea, and wheezing on auscultation of the chest after a brief prodrome of upper respiratory symptoms, as proposed by Ruuskanen and Ogra.1	Unclear/Not reported	Sweden	Europe	Consecutive	Yes	Unclear/Not reported	Unclear/Not reported	Unclear/Not reported	HRSV	Unclear/Not reported	Unclear/Not reported
Sims, 1981	1967-1968	Retrospective	8 years	8 months	8 years	Bronchiolitis	Unclear/Not reported	Unclear/Not reported	United Kingdom	Europe	Consecutive	Unclear/Not reported	Unclear/Not reported	Unclear/Not reported	skin prick and Serum tests	HRSV	Unclear/Not reported	Unclear/Not reported
Sly, 1984	1960-1978	Retrospective	Unclear/Not reported	3.2 months	16.1 years	Pneumonia	Pneumonia was diagnosed clinically and supported by chest radiographic changes compatible with adenoviral pneumonia in all cases.	Unclear/Not reported	Australia	Western Pacific	Consecutive	Yes	50	60	Unclear/Not reported	HAdV-7	Unclear/Not reported	Culture
Strannegård, 1997	Dec/1989-Apr/1990	Prospective	Unclear/Not reported	4 months	3 years	Bronchiolitis	Unclear/Not reported	Unclear/Not reported	Sweden	Europe	Consecutive	Yes	Unclear/Not reported	Unclear/Not reported	Skin prick test	HRSV	Nasopharyngeal secretions	Rapid enzyme immunoassay
