# Supplementary material for: Association of early viral lower respiratory infections and subsequent development of atopy, a systematic review and meta-analysis of cohort studies
Source: PLoS One. 2020 Apr 24;15(4):e0231816. doi: 10.1371/journal.pone.0231816 (PMC7182231; doi:10.1371/journal.pone.0231816)
Supplement: S4 Table — (PDF) [file pone.0231816.s004.pdf]

1.4. Supplementary Table 4. Risk of bias assessment

One star (1); No star (0); 0-5: High risk of bias; 6-9: Low risk of bias									
	**Selection**				**Comparability**		**Outcome**		**Risk of bias**
Author, Date	Representativeness of LRTI cohort	Selection of patients without LRTI cohort	Ascertainment of LRTI exposition	Demonstration that atopy was not present at the beginning of the study	Comparability of infants with and without LRTI in infancy age at inclusion	Comparability of infants with and without LRTI in infancy for a second important factor	Assessment of the atopy	Was follow-up long enough for the development of atopy	Complete follow up of all subjects accounted for 80%	
**Fjærli, 2005**	1	0	1	0	1	0	1	1	0	High
**García-García, 2007**	1	1	1	0	1	0	1	1	0	Low
**García-García, 2007**	1	1	1	0	1	0	1	1	0	Low
**Henderson, 2005**	1	1	1	1	1	0	1	1	1	Low
**Juntti, 2003**	1	0	1	0	1	1	1	1	0	Low
**Korppi, 2004**	1	0	1	0	1	1	1	1	1	Low
**Mikalsen, 2012**	1	1	1	0	1	1	1	1	0	Low
**Murray, 1992**	1	1	1	0	1	1	1	1	0	Low
**Nicolai, 2017**	1	1	1	0	1	1	1	1	0	Low
**Peña Zarza, 2012**	1	1	1	0	1	0	1	1	0	Low

One star (1); No star (0); 0-5: High risk of bias; 6-9: Low risk of bias									
	**Selection**				**Comparability**		**Outcome**		**Risk of bias**
Author, Date	Representativeness of LRTI cohort	Selection of patients without LRTI cohort	Ascertainment of LRTI exposition	Demonstration that atopy was not present at the beginning of the study	Comparability of infants with and without LRTI in infancy age at inclusion	Comparability of infants with and without LRTI in infancy for a second important factor	Assessment of the atopy	Was follow-up long enough for the development of atopy	Complete follow up of all subjects accounted for 80%	
**Poorisrisak, 2010**	1	1	1	0	1	0	1	1	0	High
**Poulsen, 2006**	1	1	1	0	1	1	1	1	1	Low
**Pullan, 1982**	1	1	1	0	1	1	1	1	0	Low
**Ruotsalainen, 2010**	1	1	1	0	1	1	1	1	0	Low
**Ruotsalainen, 2013**	1	1	1	0	1	1	1	1	0	Low
**Schauer, 2002**	1	1	1	0	1	1	1	1	0	Low
**Sigurs, 1995**	1	1	1	0	1	1	1	1	0	Low
**Sigurs, 1995**	1	1	1	0	1	1	1	1	0	Low
**Sigurs, 2000**	1	1	1	0	1	1	1	1	0	Low
**Sigurs, 2005**	1	1	1	0	1	1	1	1	0	Low
**Sigurs, 2010**	1	1	1	0	1	1	1	1	0	Low
**Sims, 1981**	1	1	1	0	1	1	1	1	0	Low

One star (1); No star (0); 0-5: High risk of bias; 6-9: Low risk of bias									
	**Selection**				**Comparability**		**Outcome**		**Risk of bias**
Author, Date	Representativeness of LRTI cohort	Selection of patients without LRTI cohort	Ascertainment of LRTI exposition	Demonstration that atopy was not present at the beginning of the study	Comparability of infants with and without LRTI in infancy age at inclusion	Comparability of infants with and without LRTI in infancy for a second important factor	Assessment of the atopy	Was follow-up long enough for the development of atopy	Complete follow up of all subjects accounted for 80%
**Sly, 1984**	1	1	1	0	0	1	1	1	0	Low
**Strannegård, 1997**	1	1	1	0	1	1	1	1	0	Low
