# Supplementary material for: Association of early viral lower respiratory infections and subsequent development of atopy, a systematic review and meta-analysis of cohort studies
Source: PLoS One. 2020 Apr 24;15(4):e0231816. doi: 10.1371/journal.pone.0231816 (PMC7182231; doi:10.1371/journal.pone.0231816)
Supplement: S5 Table — (PDF) [file pone.0231816.s005.pdf]

1.5. Supplementary table 5: Subgroup analyses of atopy in children with LRTI in infancy and control without respiratory diseases.

Subgroups	OR (95%CI)	95% Prediction interval	N Studies	Number LRTI +	Number LRTI -	H¶ (95%CI)	I²§ (95%CI)	P-value heterogeneity	P-value subgroup difference
**Atopy diagnosed by skin prick test**									
**Type of LRTI**									0.01
Bronchiolitis	1.7 [0.9-3]	[0.2-12.2]	11	509	7072	2.2 [1.6-2.9]	78.5 [61.9-87.8]	0	
LRTI	0.5 [0.3-1]	[0.1-4.7]	5	574	579	1.8 [1.1-2.9]	69.8 [22.8-88.2]	0.01	
**Timing**									0.014
Prospective	1.6 [0.8-3.1]	[0.1-18.1]	11	540	790	2.4 [1.9-3.2]	83.2 [71.3-90.1]	0	
Retrospective	0.6 [0.5-0.8]	[0.4-1]	5	543	6861	1 [1-1.4]	0 [0-50.8]	0.792	
**WHO Region**									0.066
Africa	0.7 [0.5-0.9]	NA	1	319	319	NA	NA	1	
Europe	1.2 [0.7-2.1]	[0.1-10.6]	15	764	7332	2.3 [1.8-2.9]	81.2 [69.9-88.2]	0	
**Age Range cases at recrutment**									0.309
< 1 year	1.1 [0.6-2]	[0.1-11.6]	13	993	7546	2.6 [2-3.2]	84.7 [75.4-90.5]	0	
< 2 years	1.7 [0.9-3.2]	[0-94.8]	3	90	105	1 [1-1.2]	0 [0-27.4]	0.867	
**Age Range cases at interview**									0.004
2-5 years	3.3 [1.9-5.6]	[1-10.7]	4	146	245	1.2 [1-1.9]	27.3 [0-72.8]	0.248	
5-10 years	0.7 [0.4-1.4]	[0.1-5.5]	8	736	7099	1.9 [1.3-2.7]	73 [44.8-86.8]	0.001	
10-15 years	0.7 [0.1-4.3]	NA	2	125	177	4.5 [2.6-7.8]	95.1 [85.3-98.4]	0	
15-20 years	1.9 [1-3.3]	NA	2	76	130	1 NA	0 NA	0.824	
**Viruses screened**									0.353
HMPV	2.1 [0.6-7.4]	NA	1	23	30	NA	NA	1	
HRSV	1.1 [0.7-1.9]	[0.1-9.6]	15	1060	7621	2.4 [1.9-3]	82.6 [72.6-89]	0	
**Positive serum test**									
**Type of LRTI**									< 0.001
Bronchiolitis	3.1 [2-4.8]	[1.2-8.1]	4	171	342	1.5 [1-2.6]	54.2 [0-84.8]	0.088	

Subgroups	OR (95%CI)	95% Prediction interval	N Studies	Number LRTI +	Number LRTI -	H[¶] (95%CI)	I^2[§] (95%CI)	P-value heterogeneity	P-value subgroup difference
LRTI	1 [0.7-1.3]	[0.2-6.1]	3	465	438	1.7 [1-3.2]	66.1 [0-90.3]	0.052	
**Timing**									0.12
Prospective	2.5 [0.9-6.5]	[0.1-87.1]	5	247	418	2.5 [1.7-3.8]	84.1 [64.3-92.9]	0	
Retrospective	1.1 [0.8-1.5]	NA	2	389	362	1 NA	5.5 NA	0.304	
**WHO Region**									0.07
Africa	1.1 [0.8-1.4]	NA	1	319	319	NA	NA	1	
Europe	2.4 [1-5.3]	[0.2-35.7]	6	317	461	2.2 [1.5-3.3]	80.2 [57-90.8]	0	
**Age Range cases at recrutment**									0.002
< 9 months	20.5 [4.4-95.9]	NA	1	42	84	NA	NA	1	
< 1 year	1.5 [0.9-2.7]	[0.3-9]	6	594	696	2 [1.3-3]	75.2 [43.8-89]	0.001	
**Age Range cases at interview**									0.004
< 2 years	20.5 [4.4-95.9]	NA	1	42	84	NA	NA	1	
5-10 years	1.1 [0.6-2.1]	[0.1-14.3]	4	509	525	1.9 [1.1-3.1]	70.9 [17-89.8]	0.016	
10-15 years	2.4 [1.1-5.2]	NA	1	44	86	NA	NA	1	
15-20 years	3.2 [1.5-7.1]	NA	1	41	85	NA	NA	1	
**Atopy diagnosis unknown not reported**									
**Type of LRTI**									0.254
Bronchiolitis	0.8 [0.4-1.7]	NA	2	50	79	1 NA	0 NA	0.79	
Pneumonia	0.4 [0.1-1.3]	NA	1	20	20	NA	NA	1	
**Timing**									0.254
Prospective	0.8 [0.4-1.7]	NA	2	50	79	1 NA	0 NA	0.79	
Retrospective	0.4 [0.1-1.3]	NA	1	20	20	NA	NA	1	
**WHO Region**									0.254
Europe	0.8 [0.4-1.7]	NA	2	50	79	1 NA	0 NA	0.79	
Western Pacific	0.4 [0.1-1.3]	NA	1	20	20	NA	NA	1	
**Age Range cases at recrutment**									0.583
< 1 year	0.6 [0.3-1.3]	NA	2	55	84	1 NA	6.7 NA	0.301	
< 2 years	1 [0.2-4.6]	NA	1	15	15	NA	NA	1	
**Age Range cases at interview**									0.503
2-5 years	1 [0.2-4.6]	NA	1	15	15	NA	NA	1	

Subgroups	OR (95%CI)	95% Prediction interval	N Studies	Number LRTI +	Number LRTI -	H¶ (95%CI)	$I^{2§}$ (95%CI)	P-value heterogeneity	P-value subgroup difference
5-10 years	0.8 [0.3-1.8]	NA	1	35	64	NA	NA	1	
15-20 years	0.4 [0.1-1.3]	NA	1	20	20	NA	NA	1	
**Viruses screened**									0.254
HAdV-7	0.4 [0.1-1.3]	NA	1	20	20	NA	NA	1	
HRSV	0.8 [0.4-1.7]	NA	2	50	79	1 NA	0 NA	0.79	
**Allergic rhinoconjunctivitis**									
**Type of LRTI**									0.021
Bronchiolitis	2.3 [1.3-3.9]	[0.6-9.3]	6	261	492	1.5 [1-2.4]	55.5 [0-82.1]	0.047	
LRTI	0.8 [0.4-1.6]	NA	2	116	115	1 NA	0 NA	0.606	
**Age Range cases at recrutment**									0.06
< 1 year	2.7 [1.2-5.9]	[0.1-65.6]	4	215	353	1.8 [1.1-3.1]	69.6 [12.5-89.4]	0.02	
< 2 years	1.1 [0.7-1.7]	[0.4-3]	4	162	254	1 [1-1]	0 [0-0]	0.953	
**Age Range cases at interview**									0.298
2-5 years	1.4 [0.5-3.6]	NA	2	55	60	1 NA	0 NA	0.993	
5-10 years	2 [0.4-10.5]	NA	2	123	169	2.5 [1.3-5.1]	84.5 [36.4-96.2]	0.011	
10-15 years	3.6 [1.6-8.1]	NA	1	46	92	NA	NA	1	
15-20 years	1.9 [0.8-4.3]	NA	2	113	247	2.4 [1.2-4.9]	82.1 [24.5-95.8]	0.018	
6. > 20 years	1 [0.4-2.4]	NA	1	40	39	NA	NA	1	
**Viruses screened**									0.39
Common respiratory viruses	1.1 [0.6-2]	NA	1	67	155	NA	NA	1	
HMPV	1.4 [0.3-5.5]	NA	1	23	30	NA	NA	1	
HRSV	2 [1.1-3.8]	[0.3-13]	6	287	422	1.7 [1.1-2.6]	64.8 [15.3-85.4]	0.014	
**Atopic dermatitis**									
**Type of LRTI**									0.292
Bronchiolitis	1.4 [0.9-2]	[0.6-3.2]	11	504	897	1.4 [1-2]	47.7 [0-73.9]	0.039	
LRTI	1 [0.6-1.6]	[0.4-2.7]	4	426	294	1 [1-2.3]	0 [0-81.4]	0.48	
**Timing**									0.284
Prospective	1.3 [0.9-1.8]	[0.7-2.5]	13	620	1053	1.3 [1-1.8]	42.8 [0-70.2]	0.051	
Retrospective	0.9 [0.4-1.7]	NA	2	310	138	1 NA	0 NA	0.453	

Subgroups	OR (95%CI)	95% Prediction interval	N Studies	Number LRTI +	Number LRTI -	H¶ (95%CI)	I²§ (95%CI)	P-value heterogeneity	P-value subgroup difference
**Age Range cases at recrutment**									0.336
< 9 months	0.8 [0.3-2.3]	NA	1	42	84	NA	NA	1	
< 1 year	1.1 [0.8-1.6]	[0.8-1.7]	9	454	713	1 [1-1.3]	0 [0-42.8]	0.766	
< 2 years	2.5 [0.8-7.6]	[0-301.4]	4	161	293	2 [1.2-3.4]	76.1 [34.2-91.3]	0.006	
< 3 years	0.7 [0.3-1.7]	NA	1	273	101	NA	NA	1	
**Age Range cases at interview**									0.498
< 2 years	1.2 [0.5-2.6]	NA	2	89	177	1.1 NA	16 NA	0.275	
2-5 years	1.2 [0.7-2.1]	[0-52.7]	3	102	153	1 [1-1.3]	0 [0-42.8]	0.834	
5-10 years	1 [0.7-1.4]	[0.6-1.6]	6	541	444	1 [1-1.8]	0 [0-68.1]	0.553	
10-15 years	1.5 [0.4-4.9]	NA	1	46	92	NA	NA	1	
15-20 years	4.9 [0.7-35.3]	NA	2	112	245	2.9 [1.5-5.7]	88.3 [55.1-96.9]	0.003	
6. > 20 years	2.2 [0.7-7.2]	NA	1	40	80	NA	NA	1	
**Viruses screened**									0.644
Common respiratory viruses	3.5 [0.3-39]	NA	2	339	254	3.8 [2.1-7]	93.2 [77.8-97.9]	0	
HMPV	1.3 [0.4-4]	NA	1	23	30	NA	NA	1	
HRSV	1.1 [0.9-1.5]	[0.8-1.6]	12	568	907	1 [1-1.2]	0 [0-30.2]	0.833	
**Pollens**									
**Type of LRTI**									0.001
Bronchiolitis	1.7 [0.9-3]	NA	2	83	172	1 NA	0 NA	0.706	
LRTI	0.2 [0-0.6]	NA	1	51	51	NA	NA	1	
**Age Range cases at interview**									0.004
5-10 years	0.2 [0-0.6]	NA	1	51	51	NA	NA	1	
10-15 years	1.5 [0.6-3.5]	NA	1	42	87	NA	NA	1	
15-20 years	1.8 [0.8-4.1]	NA	1	41	85	NA	NA	1	
**Food allergy**									
**Type of LRTI**									0.081
Bronchiolitis	1.6 [0.4-5.7]	[0-6789.9]	3	144	241	1.1 [1-3.3]	10.9 [0-90.7]	0.326	
LRTI	0.3 [0.1-1.1]	NA	1	51	51	NA	NA	1	
**Age Range cases at recrutment**									0.851

Subgroups	OR (95%CI)	95% Prediction interval	N Studies	Number LRTI +	Number LRTI -	H[¶] (95%CI)	$I^{2§}$ (95%CI)	P-value heterogeneity	P-value subgroup difference
< 1 year	1 [0.1-8.2]	NA	2	140	232	2.3 [1.1-4.7]	80.7 [17.6-95.5]	0.023	
< 2 years	0.8 [0.2-3.8]	NA	2	55	60	1 NA	0 NA	0.814	
**Age Range cases at interview**									0.081
2-5 years	1.6 [0.4-5.7]	[0-6789.9]	3	100	152	1.1 [1-3.3]	10.9 [0-90.7]	0.326	
5-10 years	0.3 [0.1-1.1]	NA	1	95	140	NA	NA	1	
**Viruses screened**									0.808
HMPV	0.6 [0.1-7.5]	NA	1	23	30	NA	NA	1	
HRSV	0.9 [0.2-3.7]	[0-738846.9]	3	172	262	1.6 [1-3]	62.1 [0-89.2]	0.072	
**Furred animals**									
**Type of LRTI**									0.021
Bronchiolitis	0.7 [0.5-0.9]	[0.5-1]	8	331	7128	1 [1-1.1]	0 [0-18.5]	0.904	
LRTI	0.1 [0-0.5]	NA	1	51	51	NA	NA	1	
**Timing**									0.959
Prospective	0.6 [0.5-0.9]	[0.4-0.9]	8	334	565	1.1 [1-1.5]	13.3 [0-56]	0.326	
Retrospective	0.6 [0.1-2.5]	NA	1	48	6614	NA	NA	1	
**Age Range cases at recrutment**									0.646
< 9 months	0.9 [0.4-2.1]	NA	1	42	84	NA	NA	1	
< 1 year	0.6 [0.5-0.9]	[0.4-1]	6	285	7035	1.2 [1-1.9]	29.5 [0-71.2]	0.214	
< 2 years	0.5 [0.2-1.1]	NA	2	55	60	1 NA	0 NA	0.735	
**Age Range cases at interview**									0.958
< 2 years	0.7 [0.4-1.2]	NA	2	89	177	1 NA	0 NA	0.431	
2-5 years	0.5 [0.2-1.1]	NA	2	55	60	1 NA	0 NA	0.735	
5-10 years	0.5 [0.2-1.4]	[0-20881.2]	3	146	6758	1.8 [1-3.4]	70.6 [0-91.4]	0.034	
10-15 years	0.6 [0.3-1.3]	NA	1	46	92	NA	NA	1	
15-20 years	0.7 [0.3-1.4]	NA	1	46	92	NA	NA	1	
**Viruses screened**									0.849
HMPV	0.6 [0.2-1.8]	NA	1	23	30	NA	NA	1	
HRSV	0.6 [0.5-0.9]	[0.4-0.9]	8	359	7149	1.1 [1-1.5]	13 [0-55.6]	0.329	
**House dust mite**									

Subgroups	OR (95%CI)	95% Prediction interval	N Studies	Number LRTI +	Number LRTI -	H[¶] (95%CI)	I^2[§] (95%CI)	P-value heterogeneity	P-value subgroup difference
**Type of LRTI**									0.102
Bronchiolitis	1.3 [0.7-2.5]	[0.1-10.8]	4	204	6910	1.4 [1-2.4]	46.1 [0-82.1]	0.135	
LRTI	0.1 [0-2]	NA	1	51	51	NA	NA	1	
**Timing**									0.071
Prospective	1.6 [0.8-2.9]	[0.4-6]	4	206	296	1.3 [1-2.2]	41.1 [0-80.1]	0.165	
Retrospective	0.5 [0.1-1.5]	NA	1	49	6665	NA	NA	1	
**Age Range cases at interview**									0.046
5-10 years	0.6 [0.2-1.3]	[0-123.1]	3	173	6789	1 [1-3.2]	7.3 [0-90.4]	0.34	
10-15 years	1.5 [0.5-4.4]	NA	1	42	87	NA	NA	1	
15-20 years	2.8 [1-7.6]	NA	1	40	85	NA	NA	1	
**Positive serum test for food**									
**Age Range cases at recrutment**									0.019
< 9 months	41.5 [5.2-330]	NA	1	42	84	NA	NA	1	
< 1 year	3 [1.4-6.3]	NA	2	88	179	1.2 NA	34.2 NA	0.218	
**Age Range cases at interview**									0.03
< 2 years	41.5 [5.2-330]	NA	1	42	84	NA	NA	1	
2-5 years	5.1 [1.6-16.1]	NA	1	44	92	NA	NA	1	
5-10 years	2 [0.7-5.3]	NA	1	44	87	NA	NA	1	
**Positive serum test for inhalants**									
**Age Range cases at recrutment**									0.99
< 9 months	4.2 [0.4-47.1]	NA	1	42	84	NA	NA	1	
< 1 year	4.2 [2-8.8]	NA	2	88	179	1.5 [1-3]	55.2 [0-89.1]	0.135	
**Age Range cases at interview**									0.327
< 2 years	4.2 [0.4-47.1]	NA	1	42	84	NA	NA	1	
2-5 years	9.9 [2.6-37.7]	NA	1	44	92	NA	NA	1	
5-10 years	2.9 [1.2-7]	NA	1	44	87	NA	NA	1	

CI: confidence interval; OR: Odds ratio; NA: not applicable.
