# Supplementary material for: Association of early viral lower respiratory infections and subsequent development of atopy, a systematic review and meta-analysis of cohort studies
Source: PLoS One. 2020 Apr 24;15(4):e0231816. doi: 10.1371/journal.pone.0231816 (PMC7182231; doi:10.1371/journal.pone.0231816)
Supplement: S6 Table — (PDF) [file pone.0231816.s006.pdf]

1.6. Supplementary table 6. Metaregression analyses for the association of LRTI with subsequent atopy

Subgroup	Univariate Model		Multivariate Model		
	P-Value	OR (95% CI)	P-Value	OR (95% CI)	R2
**Atopy diagnosed by skin prick test**					
**Type of LRTI**					
Bronchiolitis		1		1	
LRTI	0,017	0,31 [0,12-0,81]	0,017	0,31 [0,12-0,81]	31,31%
**Timing**					
Prospective		1			
Retrospective	0,059	0,38 [0,14-1,04]			
**WHO Region**					
Africa		1			
Europe	0,536	1,82 [0,27-12,04]			
**Viruses screened**					
HMPV		1			
HRSV	0,576	0,53 [0,06-5]			
**Follow-up delay (years)**	0,704	0,98 [0,89-1,08]			
**Positive serum test**					
**Type of LRTI**					
Bronchiolitis		1		1	
LRTI	< 0.001	0,31 [0,19-0,52]	< 0.001	0,31 [0,19-0,52]	100%
**Timing**					
Prospective		1			
Retrospective	0,454	0,57 [0,13-2,45]			
**WHO Region**					

Subgroup	Univariate Model		Multivariate Model		
	P-Value	OR (95% CI)	P-Value	OR (95% CI)	R2
Africa		1			
Europe	0,357	2,19 [0,41-11,57]			
**Follow-up delay (years)**	0,742	0,98 [0,84-1,13]			
**Atopy diagnosis unknown not reported**					
**Type of LRTI**					
Bronchiolitis		1			
Pneumonia	0,254	0,42 [0,09-1,86]			
**Timing**					
Prospective		1			
Retrospective	0,254	0,42 [0,09-1,86]			
**WHO Region**					
Europe		1			
Western Pacific	0,254	0,42 [0,09-1,86]			
**Viruses screened**					
HAdV-7		1			
HRSV	0,254	2,38 [0,54-10,55]			
**Follow-up delay (years)**	0,248	0,92 [0,81-1,06]			
**Allergic rhinoconjunctivitis**					
**Type of LRTI**					
Bronchiolitis		1		1	
LRTI	0,037	0,37 [0,14-0,94]	0,037	0,37 [0,14-0,94]	57,51%
**Viruses screened**					
Common respiratory viruses		1			
HRSV	0,294	1,85 [0,59-5,84]			

Subgroup	Univariate Model		Multivariate Model		
	P-Value	OR (95% CI)	P-Value	OR (95% CI)	R2
**Follow-up delay (years)**	0,767	0,99 [0,92-1,06]			
**Atopic dermatitis**					
**Type of LRTI**					
Bronchiolitis		1			
LRTI	0,322	0,76 [0,44-1,31]			
**Timing**		1			
Prospective					
Retrospective	0,314	0,68 [0,32-1,44]			
**Viruses screened**					
Common respiratory viruses		1			
HRSV	0,259	0,59 [0,24-1,47]			
**Follow-up delay (years)**	0,059	1,04 [1-1,09]			
**Pollens**					
**Type of LRTI**					
Bronchiolitis		1		1	
LRTI	0,001	0,09 [0,02-0,38]	0,001	0,09 [0,02-0,38]	100%
**Follow-up delay (years)**	0,007	1,22 [1,05-1,4]			
**Food allergy**					
**Type of LRTI**					
Bronchiolitis		1			
LRTI	0,081	0,19 [0,03-1,23]			

Subgroup		Univariate Model		Multivariate Model	
	P-Value	OR (95% CI)	P-Value	OR (95% CI)	R2
**Viruses screened**					
HMPV		1			
HRSV	0,854	1,33 [0,07-26,68]			
**Follow-up delay (years)**	0,053	0,63 [0,4-1,01]			
**Furred animals**					
**Type of LRTI**		1			
Bronchiolitis					
LRTI	0,021	0,16 [0,03-0,76]			
**Timing**		1			
Prospective					
Retrospective	0,959	0,96 [0,23-4,11]			
**Viruses screened**					
HMPV		1			
HRSV	0,849	1,12 [0,34-3,67]			
**Follow-up delay (years)**	0,912	1 [0,96-1,05]			
**House dust mite**					
**Type of LRTI**		1			
Bronchiolitis					
LRTI	0,110	0,08 [0-1,77]			
**Timing**		1			
Prospective					
Retrospective	0,071	0,3 [0,08-1,11]			
**Follow-up delay (years)**	0,014	1,12 [1,02-1,22]	0,014	1,12 [1,02-1,22]	100%

Subgroup	Univariate Model		Multivariate Model		
	P-Value	OR (95% CI)	P-Value	OR (95% CI)	R2
**Positive serum test for food**					
**Follow-up delay (years)**	0,016	0,66 [0,47-0,92]	0,016	0,66 [0,47-0,92]	100%
**Positive serum test for inhalants**					
**Follow-up delay (years)**	0,246	0,81 [0,57-1,16]			
