# Supplementary material for: Association of early viral lower respiratory infections and subsequent development of atopy, a systematic review and meta-analysis of cohort studies
Source: PLoS One. 2020 Apr 24;15(4):e0231816. doi: 10.1371/journal.pone.0231816 (PMC7182231; doi:10.1371/journal.pone.0231816)
Supplement: S7 Table — (PDF) [file pone.0231816.s007.pdf]

1.7. Supplementary table 7. P-value of Khi-2 and Fisher exact tests for qualitative confounding factors

Author, year	Confounding factor	Data extracted from included studies				Results from this study		
		Total LRTI +	Number in LRTI +	Total LRTI -	Number in LRTI -	P-value Khi-2 test	P-value Fisher exact test	Status
García-García, 2007	Asthma in father	23	3	30	4	1	1	Symmetric
García-García, 2007	Asthma in father	32	5	30	4	1	1	Symmetric
Juntti, 2003	Asthma in father	73	8	74	5	0,54	0,4	Symmetric
García-García, 2007	Asthma in mother	23	6	30	6	0,85	0,74	Symmetric
García-García, 2007	Asthma in mother	32	6	30	6	1	1	Symmetric
Juntti, 2003	Asthma in mother	76	12	76	4	0,06	0,06	Symmetric
Nicolai, 2017	Asthma in parents	273	49	101	11	0,14	0,11	Symmetric
Ruotsalainen, 2010	Asthma in parents	40	11	80	11	0,11	0,08	Symmetric
Schauer, 2002	Asthma in parents	42	4	84	5	0,71	0,48	Symmetric
Sigurs, 1995	Asthma in parents	47	0	93	3	0,53	0,55	Symmetric
Sigurs, 2000	Asthma in parents	47	16	93	19	0,12	0,1	Symmetric
Sigurs, 2005	Asthma in parents	46	17	92	25	0,33	0,25	Symmetric

Author, year	Confounding factor	Data extracted from included studies				Results from this study		
		Total LRTI +	Number in LRTI +	Total LRTI -	Number in LRTI -	P-value Khi-2 test	P-value Fisher exact test	Status
Sigurs, 2010	Asthma in parents	46	18	92	25	0,22	0,18	Symmetric
García-García, 2007	Asthma in siblings	23	8	30	4	0,13	0,1	Symmetric
García-García, 2007	Asthma in siblings	32	5	30	4	1	1	Symmetric
Ruotsalainen, 2010	Asthma in siblings	39	11	39	11	1	1	Symmetric
Ruotsalainen, 2013	Asthma in siblings	65	18	154	24	0,06	0,06	Symmetric
García-García, 2007	Atopy in siblings	23	6	30	3	0,24	0,15	Symmetric
Juntti, 2003	Atopy in siblings	76	42	76	40	0,87	0,87	Symmetric
Nicolai, 2017	Atopy in siblings	273	22	101	9	0,96	0,83	Symmetric
Murray, 1992	Birth weight <2500 g	73	13	73	7	0,23	0,23	Symmetric
Poulsen, 2006	Birth weight <2500 g	175	24	175	21	0,75	0,75	Symmetric
Fjærli, 2005	Boys	22	12	64	36	1	1	Symmetric
García-García, 2007	Boys	23	14	30	13	0,32	0,27	Symmetric
García-García, 2007	Boys	32	17	30	13	0,61	0,46	Symmetric
Mikalsen, 2012	Boys	90	49	141	85	0,46	0,41	Symmetric
Nicolai, 2017	Boys	273	167	101	59	0,72	0,64	Symmetric
Peña Zarza, 2012	Boys	15	11	15	8	0,45	0,45	Symmetric
Ruotsalainen, 2013	Boys	67	45	155	104	1	1	Symmetric
Schauer, 2002	Boys	42	18	84	36	1	1	Symmetric
Sigurs, 1995	Boys	47	21	93	42	1	1	Symmetric
Sigurs, 2000	Boys	47	21	93	42	1	1	Symmetric
Sigurs, 2005	Boys	46	20	92	41	1	1	Symmetric
Sly, 1984	Boys	20	10	20	12	0,75	0,75	Symmetric
Fjærli, 2005	Current smoke exposure	35	19	64	28	0,43	0,4	Symmetric
Nicolai, 2017	Current smoke	273	126	101	35	0,06	0,06	Symmetric

Author, year	Confounding factor	Data extracted from included studies				Results from this study		
		Total LRTI +	Number in LRTI +	Total LRTI -	Number in LRTI -	P-value Khi-2 test	P-value Fisher exact test	Status
	exposure							
Peña Zarza, 2012	Current smoke exposure	15	8	15	3	0,13	0,13	Symmetric
Poulsen, 2006	Current smoke exposure	323	68	323	70	0,92	0,92	Symmetric
Sigurs, 2000	Current smoke exposure	47	20	93	36	0,8	0,72	Symmetric
Sigurs, 2005	Current smoke exposure	46	16	92	39	0,5	0,46	Symmetric
Sigurs, 2010	Current smoke exposure	45	10	92	14	0,44	0,34	Symmetric
Sly, 1984	Current smoke exposure	20	12	20	14	0,74	0,74	Symmetric
Juntti, 2003	Day care attendance	76	21	76	16	0,45	0,45	Symmetric
Murray, 1992	Family history of asthma	73	18	73	18	1	1	Symmetric
Poulsen, 2006	Family history of asthma	317	74	317	57	0,12	0,12	Symmetric
Schauer, 2002	Family history of asthma	42	9	84	13	0,56	0,46	Symmetric
Sigurs, 2000	Family history of asthma	47	21	93	27	0,1	0,09	Symmetric
Sigurs, 2005	Family history of asthma	46	23	92	32	0,12	0,1	Symmetric
Sigurs, 2010	Family history of asthma	46	24	92	32	0,08	0,07	Symmetric
Strannegård, 1997	Family history of asthma	47	0	93	3	0,53	0,55	Symmetric

Author, year	Confounding factor	Data extracted from included studies				Results from this study		
		Total LRTI +	Number in LRTI +	Total LRTI -	Number in LRTI -	P-value Khi-2 test	P-value Fisher exact test	Status
Juntti, 2003	Family history of atopy	76	59	76	60	1	1	Symmetric
Poulsen, 2006	Family history of atopy	317	74	317	57	0,12	0,12	Symmetric
Sigurs, 2010	Family history of atopy	46	34	92	68	1	1	Symmetric
Sims, 1981	Family history of atopy	32	16	26	10	0,54	0,43	Symmetric
Schauer, 2002	Heredity for atopy	42	17	84	49	0,09	0,09	Symmetric
Sigurs, 1995	Heredity for atopy	47	18	93	34	0,99	0,86	Symmetric
Sigurs, 2000	Heredity for atopy	47	33	93	60	0,63	0,57	Symmetric
Sigurs, 2005	Heredity for atopy	46	34	92	68	1	1	Symmetric
Strannegård, 1997	Heredity for atopy	47	9	93	18	1	1	Symmetric
García-García, 2007	Maternal atopy	23	7	30	6	0,58	0,52	Symmetric
García-García, 2007	Maternal atopy	32	13	30	6	0,14	0,1	Symmetric
Juntti, 2003	Maternal atopy	76	35	76	29	0,41	0,41	Symmetric
García-García, 2007	Maternal smoking	23	7	30	12	0,67	0,57	Symmetric
García-García, 2007	Maternal smoking	32	21	30	12	0,08	0,07	Symmetric
Murray, 1992	Maternal smoking	73	40	73	41	1	1	Symmetric
Juntti, 2003	Maternal smoking during pregnancy	76	8	76	14	0,25	0,25	Symmetric
Nicolai, 2017	Maternal smoking during pregnancy	273	33	101	16	0,43	0,39	Symmetric
Murray, 1992	No (% breast fed)	73	30	73	27	0,73	0,74	Symmetric
Schauer, 2002	Parental atopy	42	6	84	9	0,77	0,57	Symmetric
Sigurs, 1995	Parental atopy	47	9	93	18	1	1	Symmetric
Sigurs, 2000	Parental atopy	47	29	93	45	0,19	0,15	Symmetric
Sigurs, 2005	Parental atopy	46	28	92	50	0,59	0,59	Symmetric
Sigurs, 2010	Parental atopy	46	30	92	52	0,43	0,36	Symmetric

Author, year	Confounding factor	Data extracted from included studies				Results from this study		
		Total LRTI +	Number in LRTI +	Total LRTI -	Number in LRTI -	P-value Khi-2 test	P-value Fisher exact test	Status
García-García, 2007	Paternal atopy	23	6	30	5	0,62	0,5	Symmetric
García-García, 2007	Paternal atopy	32	4	30	5	0,92	0,73	Symmetric
Juntti, 2003	Paternal atopy	73	17	74	24	0,29	0,27	Symmetric
García-García, 2007	Paternal smoking	23	8	30	10	1	1	Symmetric
García-García, 2007	Paternal smoking	32	18	30	10	0,12	0,08	Symmetric
Murray, 1992	Paternal smoking	73	35	73	39	0,62	0,62	Symmetric
Juntti, 2003	Pets at home	76	31	76	41	0,14	0,14	Symmetric
Poulsen, 2006	Pets at home	328	96	328	76	0,09	0,09	Symmetric
García-García, 2007	Prematurity	32	6	30	3	0,54	0,48	Symmetric
Murray, 1992	Prematurity	73	7	73	7	1	1	Symmetric
Fjærli, 2005	Siblings in the house	35	11	64	26	0,49	0,39	Symmetric
Poulsen, 2006	Siblings in the house	317	188	317	170	0,17	0,17	Symmetric
Juntti, 2003	Smoke exposure	76	32	76	25	0,32	0,32	Symmetric
Sigurs, 1995	Smoke exposure	47	21	93	42	1	1	Symmetric
Sigurs, 2000	Smoke exposure	47	24	93	49	1	0,86	Symmetric
Sly, 1984	Smoke exposure	20	3	20	3	1	1	Symmetric
