# Supplementary material for: Association of early viral lower respiratory infections and subsequent development of atopy, a systematic review and meta-analysis of cohort studies
Source: PLoS One. 2020 Apr 24;15(4):e0231816. doi: 10.1371/journal.pone.0231816 (PMC7182231; doi:10.1371/journal.pone.0231816)
Supplement: S8 Table — (PDF) [file pone.0231816.s008.pdf]

1.8. Supplementary table 8. Sensitivity analyses of the symmetrically distributed confounding factors

Subgroups	OR (95%CI)	95% Prediction interval	N Studies	Number LRTI +	Number LRTI -	H (95%CI)	I^2 (95%CI)	P heterogeneity
**Positive serum test**								
Family history of asthma	2.6 [1.3-5.1]	[0.3-26.6]	5	490	661	2.4 [1.6-3.6]	82.4 [59.6-92.3]	< 0.001

Subgroups	OR (95%CI)	95% Prediction interval	N Studies	Number LRTI +	Number LRTI -	H (95%CI)	I^2 (95%CI)	P heterogeneity
Asthma in parents	3.1 [2-4.8]	[1.2-8.1]	4	171	342	1.5 [1-2.6]	54.2 [0-84.8]	0,088
Asthma in father	0.4 [0.2-0.9]	NA	1	76	76	NA	NA	1
Asthma in mother	0.4 [0.2-0.9]	NA	1	76	76	NA	NA	1
Family history of atopy	1.1 [0.5-2.7]	[0-41667.8]	3	436	480	2.6 [1.5-4.4]	84.7 [54.7-94.9]	0,001
Heredity for atopy	3.6 [1.7-7.8]	[0-8756.3]	3	130	257	1.8 [1-3.3]	69.4 [0-91.1]	0,038
Atopy in siblings	0.4 [0.2-0.9]	NA	1	76	76	NA	NA	1
Parental atopy	3.1 [2-4.8]	[1.2-8.1]	4	171	342	1.5 [1-2.6]	54.2 [0-84.8]	0,088
Maternal atopy	0.4 [0.2-0.9]	NA	1	76	76	NA	NA	1
Paternal atopy	0.4 [0.2-0.9]	NA	1	76	76	NA	NA	1
Pets at home	0.8 [0.5-1.4]	NA	2	395	395	2.1 [1-4.4]	77.4 [0.9-94.8]	0,036
Boys	3.6 [1.7-7.8]	[0-8756.3]	3	130	257	1.8 [1-3.3]	69.4 [0-91.1]	0,038
Current smoke exposure	1.9 [1.1-3.1]	[0.3-14.1]	4	448	577	2 [1.2-3.3]	74 [27.2-90.7]	0,009
Smoke exposure	1 [0.3-3.4]	NA	2	120	163	3 [1.6-5.9]	89.1 [59.3-97.1]	0,002
Maternal smoking during pregnancy	0.4 [0.2-0.9]	NA	1	76	76	NA	NA	1
Siblings in the house	1.1 [0.8-1.4]	NA	1	319	319	NA	NA	1
Day care attendance	0.4 [0.2-0.9]	NA	1	76	76	NA	NA	1
Birth weight inf2500 g	1.1 [0.8-1.4]	NA	1	319	319	NA	NA	1
**Allergic rhinoconjunctivitis**								
Family history of asthma	4 [2.3-6.8]	[0.1-129.4]	3	139	277	1 [1-2]	0 [0-74.5]	0,665
Asthma in siblings	1.2 [0.7-2]	[0-32.6]	3	122	215	1 [1-1]	0 [0-0]	0,911
Asthma in parents	2.8 [1.4-5.4]	[0.2-32.6]	4	179	316	1.6 [1-2.8]	61.7 [0-87.2]	0,049
Asthma in father	1 [0.5-2]	[0-87.9]	3	131	136	1 [1-2.3]	0 [0-80.7]	0,584
Asthma in mother	1 [0.5-2]	[0-87.9]	3	131	136	1 [1-2.3]	0 [0-80.7]	0,584
Family history of atopy	1.7 [0.5-5.4]	NA	2	122	168	2.6 [1.3-5.2]	84.8 [38.2-96.3]	0,01
Heredity for atopy	4.2 [2-8.8]	NA	2	93	185	1 NA	0 NA	0,387
Atopy in siblings	0.9 [0.4-2]	NA	2	99	106	1 NA	0 NA	0,405
Parental atopy	4 [2.3-6.8]	[0.1-129.4]	3	139	277	1 [1-2]	0 [0-74.5]	0,665

Subgroups	OR (95%CI)	95% Prediction interval	N Studies	Number LRTI +	Number LRTI -	H (95%CI)	I² (95%CI)	P heterogeneity
Maternal atopy	1 [0.5-2]	[0-87.9]	3	131	136	1 [1-2.3]	0 [0-80.7]	0,584
Paternal atopy	1 [0.5-2]	[0-87.9]	3	131	136	1 [1-2.3]	0 [0-80.7]	0,584
Pets at home	0.7 [0.2-1.9]	NA	1	76	76	NA	NA	1
Boys	1.7 [1-2.9]	[0.5-6.5]	6	255	439	1.4 [1-2.3]	51.6 [0-80.7]	0,066
Current smoke exposure	2.8 [1.4-5.4]	[0.2-32.6]	4	179	316	1.6 [1-2.8]	61.7 [0-87.2]	0,049
Smoke exposure	2 [0.4-10.5]	NA	2	123	169	2.5 [1.3-5.1]	84.5 [36.4-96.2]	0,011
Maternal smoking during pregnancy	0.7 [0.2-1.9]	NA	1	76	76	NA	NA	1
Maternal smoking	1.4 [0.5-3.6]	NA	2	55	60	1 NA	0 NA	0,993
Paternal smoking	1.4 [0.5-3.6]	NA	2	55	60	1 NA	0 NA	0,993
Day care attendance	0.7 [0.2-1.9]	NA	1	76	76	NA	NA	1
Prematurity	1.4 [0.4-5]	NA	1	32	30	NA	NA	1
**Furred animals**								
Family history of asthma	0.8 [0.5-1.1]	[0.3-1.8]	4	181	361	1 [1-1.6]	0 [0-60.6]	0,761
Asthma in siblings	0.5 [0.2-1.1]	NA	2	55	60	1 NA	0 NA	0,735
Asthma in parents	0.8 [0.5-1.1]	[0.3-1.8]	4	181	361	1 [1-1.6]	0 [0-60.6]	0,761
Asthma in father	0.4 [0.2-0.7]	[0-33.7]	3	106	111	1.2 [1-2.2]	35.4 [0-79.2]	0,213
Asthma in mother	0.4 [0.2-0.7]	[0-33.7]	3	106	111	1.2 [1-2.2]	35.4 [0-79.2]	0,213
Family history of atopy	0.4 [0.1-1.2]	NA	2	97	143	2.2 [1-4.5]	78.6 [6.8-95.1]	0,031
Heredity for atopy	0.8 [0.5-1.3]	[0-14.8]	3	135	269	1 [1-2.2]	0 [0-80]	0,595
Atopy in siblings	0.3 [0.1-0.9]	NA	2	74	81	1.7 [1-3.6]	65.5 [0-92.2]	0,089
Parental atopy	0.8 [0.5-1.1]	[0.3-1.8]	4	181	361	1 [1-1.6]	0 [0-60.6]	0,761
Maternal atopy	0.4 [0.2-0.7]	[0-33.7]	3	106	111	1.2 [1-2.2]	35.4 [0-79.2]	0,213
Paternal atopy	0.4 [0.2-0.7]	[0-33.7]	3	106	111	1.2 [1-2.2]	35.4 [0-79.2]	0,213
Pets at home	0.1 [0-0.5]	NA	1	51	51	NA	NA	1
Boys	0.7 [0.5-1.1]	[0.4-1.4]	5	190	329	1 [1-1.7]	0 [0-64.4]	0,674
Current smoke exposure	0.8 [0.5-1.2]	[0.1-11.5]	3	139	277	1 [1-2.3]	0 [0-81]	0,578
Smoke exposure	0.4 [0.1-1.9]	NA	2	98	144	2.6 [1.3-5.2]	85.2 [40.2-96.4]	0,009

Subgroups	OR (95%CI)	95% Prediction interval	N Studies	Number LRTI +	Number LRTI -	H (95%CI)	I² (95%CI)	P heterogeneity
Maternal smoking during pregnancy	0.1 [0-0.5]	NA	1	51	51	NA	NA	1
Maternal smoking	0.5 [0.2-1.1]	NA	2	55	60	1 NA	0 NA	0,735
Paternal smoking	0.5 [0.2-1.1]	NA	2	55	60	1 NA	0 NA	0,735
Day care attendance	0.1 [0-0.5]	NA	1	51	51	NA	NA	1
Prematurity	0.4 [0.1-1.3]	NA	1	32	30	NA	NA	1
**Positive serum test for food**								
Family history of asthma	6.7 [0.9-51.3]	NA	2	86	171	2.6 [1.3-5.2]	85.2 [39.9-96.3]	0,009
Asthma in parents	5.3 [1.7-16.7]	[0-1015892.4]	3	130	263	1.9 [1-3.5]	71.5 [3.4-91.6]	0,03
Heredity for atopy	5.3 [1.7-16.7]	[0-1015892.4]	3	130	263	1.9 [1-3.5]	71.5 [3.4-91.6]	0,03
Parental atopy	5.3 [1.7-16.7]	[0-1015892.4]	3	130	263	1.9 [1-3.5]	71.5 [3.4-91.6]	0,03
Boys	5.3 [1.7-16.7]	[0-1015892.4]	3	130	263	1.9 [1-3.5]	71.5 [3.4-91.6]	0,03
Current smoke exposure	2 [0.7-5.3]	NA	1	44	87	NA	NA	1
Smoke exposure	3 [1.4-6.3]	NA	2	88	179	1.2 NA	34.2 NA	0,218
**Positive serum test for inhalants**								
Family history of asthma	3 [1.3-6.9]	NA	2	86	171	1 NA	0 NA	0,789
Asthma in parents	4.2 [2.1-8.5]	[0-402.8]	3	130	263	1.1 [1-3.3]	10.5 [0-90.7]	0,327
Heredity for atopy	4.2 [2.1-8.5]	[0-402.8]	3	130	263	1.1 [1-3.3]	10.5 [0-90.7]	0,327
Parental atopy	4.2 [2.1-8.5]	[0-402.8]	3	130	263	1.1 [1-3.3]	10.5 [0-90.7]	0,327
Boys	4.2 [2.1-8.5]	[0-402.8]	3	130	263	1.1 [1-3.3]	10.5 [0-90.7]	0,327
Current smoke exposure	2.9 [1.2-7]	NA	1	44	87	NA	NA	1
Smoke exposure	4.2 [2-8.8]	NA	2	88	179	1.5 [1-3]	55.2 [0-89.1]	0,135
