# Supplementary material for: Association of early viral lower respiratory infections and subsequent development of atopy, a systematic review and meta-analysis of cohort studies
Source: PLoS One. 2020 Apr 24;15(4):e0231816. doi: 10.1371/journal.pone.0231816 (PMC7182231; doi:10.1371/journal.pone.0231816)
Supplement: S1 Fig — (PDF) [file pone.0231816.s009.pdf]

2.1. Supplementary Figure 1. Forest plot of the Comparison of atopy in people with and without LRTI in infancy
