# Supplementary figures and images for: Association of early viral lower respiratory infections and subsequent development of atopy, a systematic review and meta-analysis of cohort studies
Source: PLoS One. 2020 Apr 24;15(4):e0231816. doi: 10.1371/journal.pone.0231816 (PMC7182231; doi:10.1371/journal.pone.0231816)

2.2. Supplementary Figure 2. Funnel plot for publication for atopy diagnosed by skin prick tests

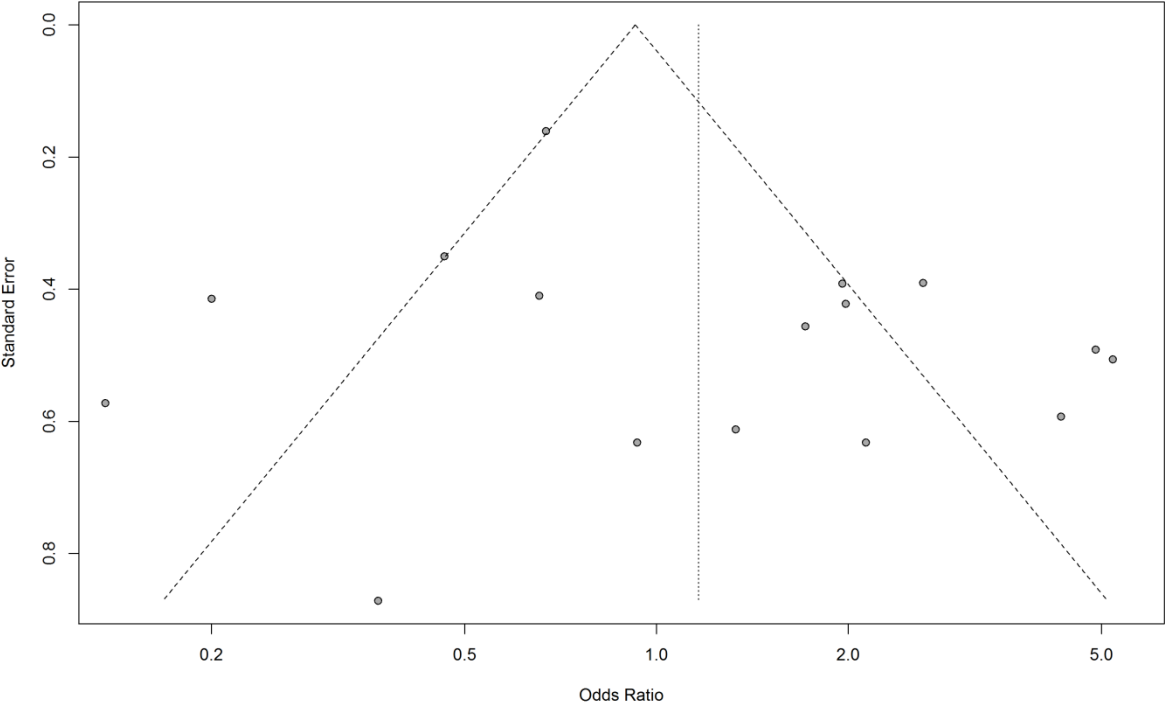

Supplement: S2 Fig — (PDF) [file pone.0231816.s010.pdf]

2.3. Supplementary Figure 3. Funnel plot for publication for atopy diagnosed by serum tests

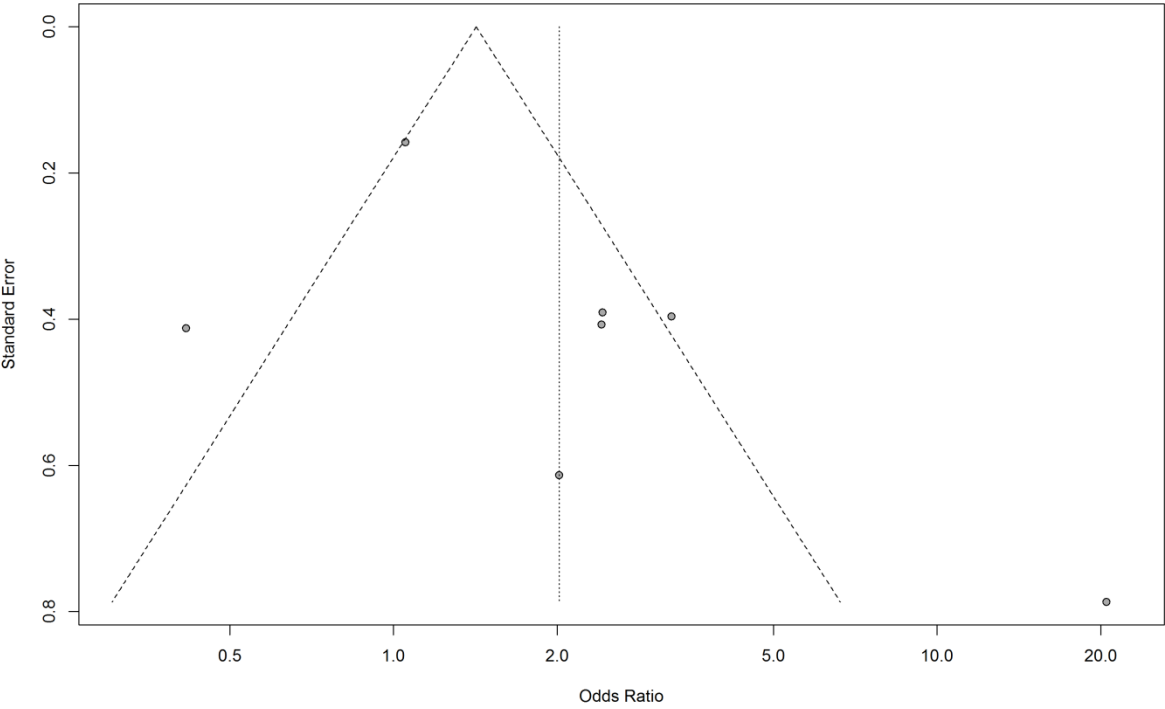

Supplement: S3 Fig — (PDF) [file pone.0231816.s011.pdf]

2.4. Supplementary Figure 4. Funnel plot for publication for atopy diagnosis unknown/not reported

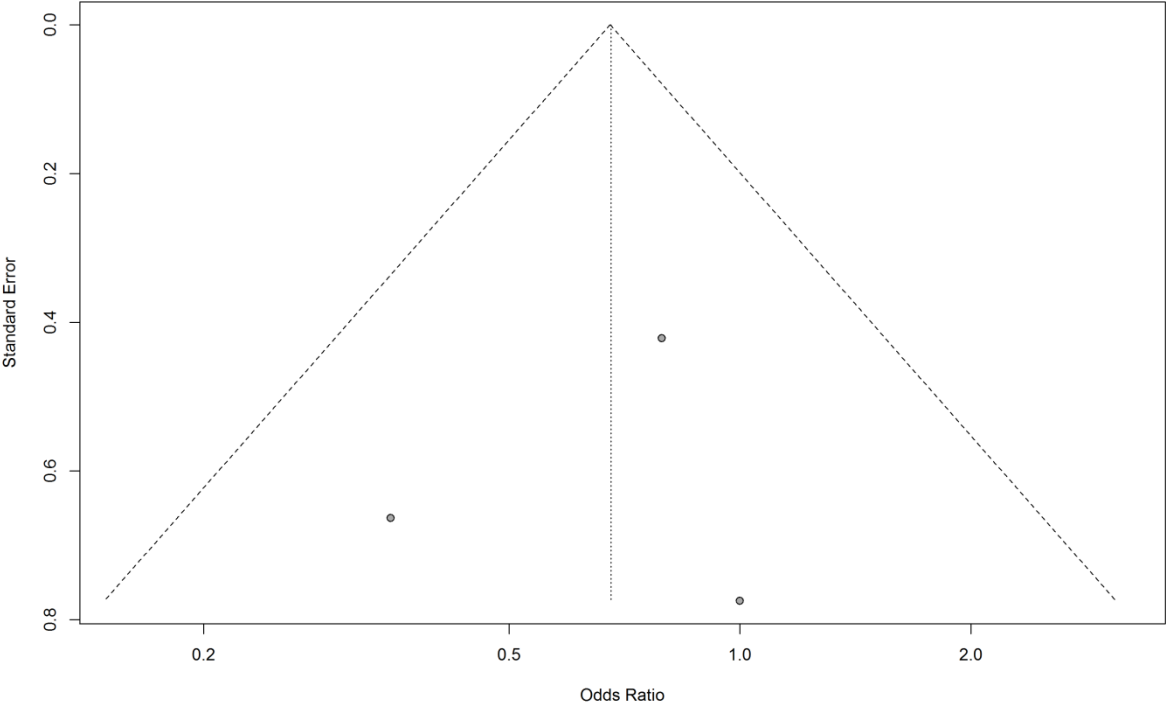

Supplement: S4 Fig — (PDF) [file pone.0231816.s012.pdf]

2.5. Supplementary Figure 5. Funnel plot for publication for allergic rhinoconjunctivitis

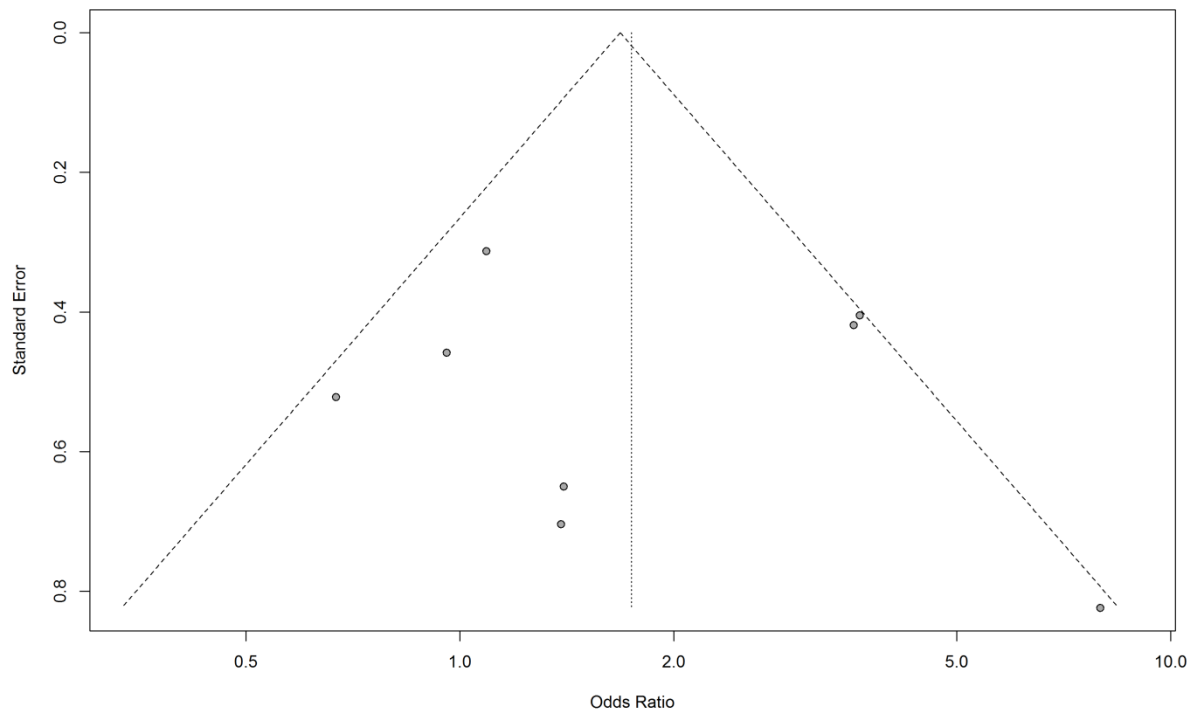

Supplement: S5 Fig — (PDF) [file pone.0231816.s013.pdf]

2.6. Supplementary Figure 6. Funnel plot for publication for atopic dermatitis

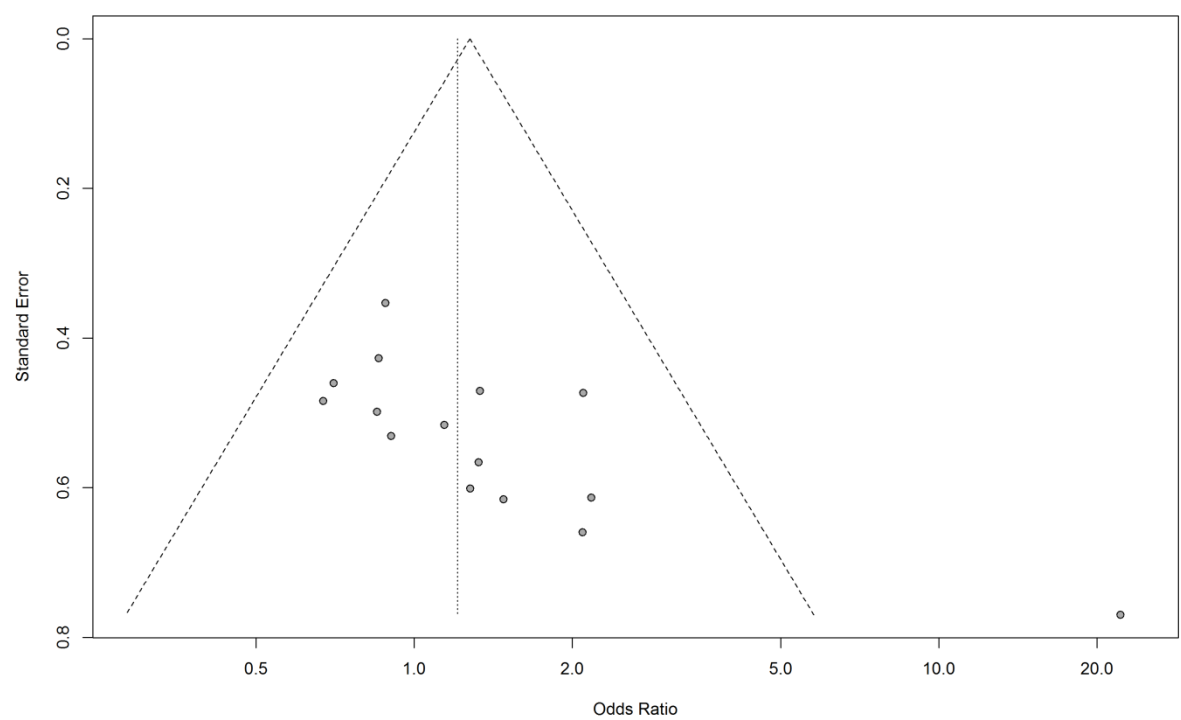

Supplement: S6 Fig — (PDF) [file pone.0231816.s014.pdf]

2.7. Supplementary Figure 7. Funnel plot for publication for pollens

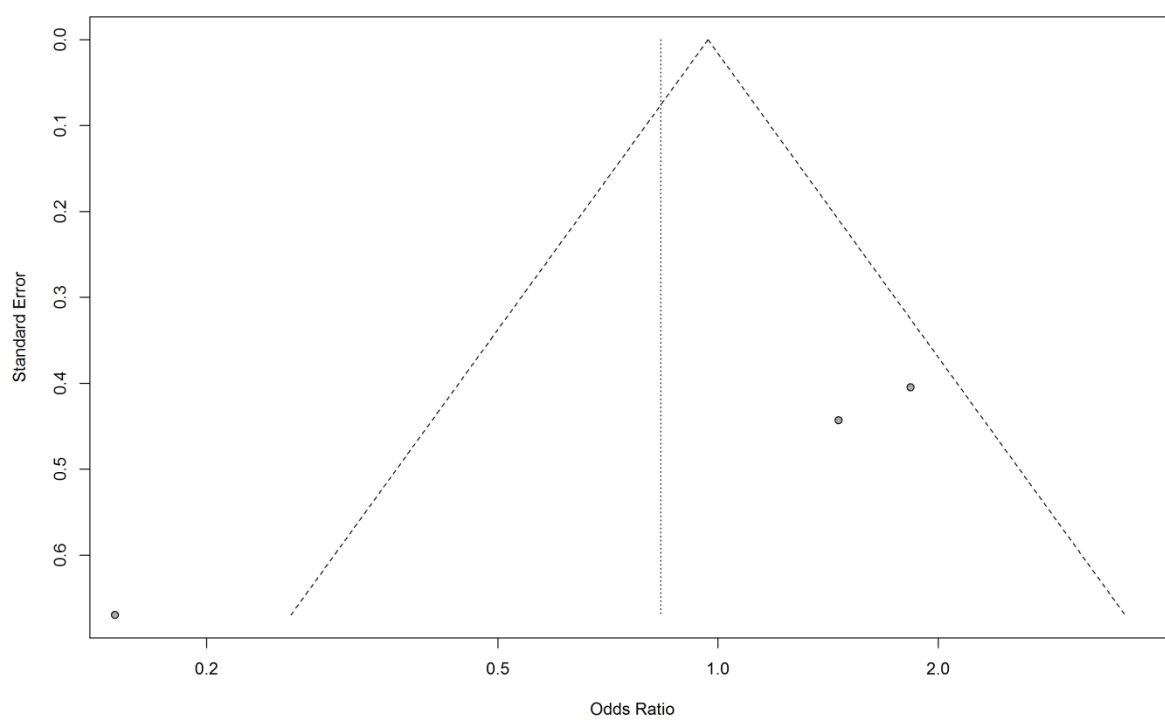

Supplement: S7 Fig — (PDF) [file pone.0231816.s015.pdf]

2.8. Supplementary Figure 8. Funnel plot for publication for food allergy

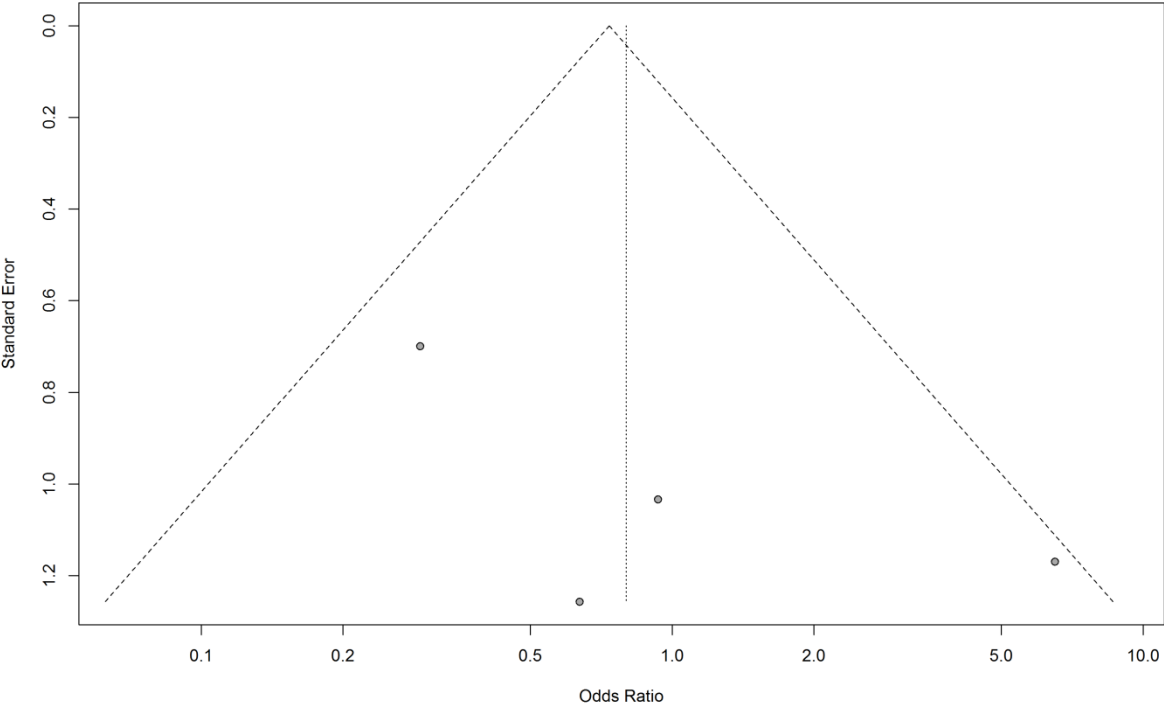

Supplement: S8 Fig — (PDF) [file pone.0231816.s016.pdf]

## 2.9. Supplementary Figure 9. Funnel plot for publication for furred animals

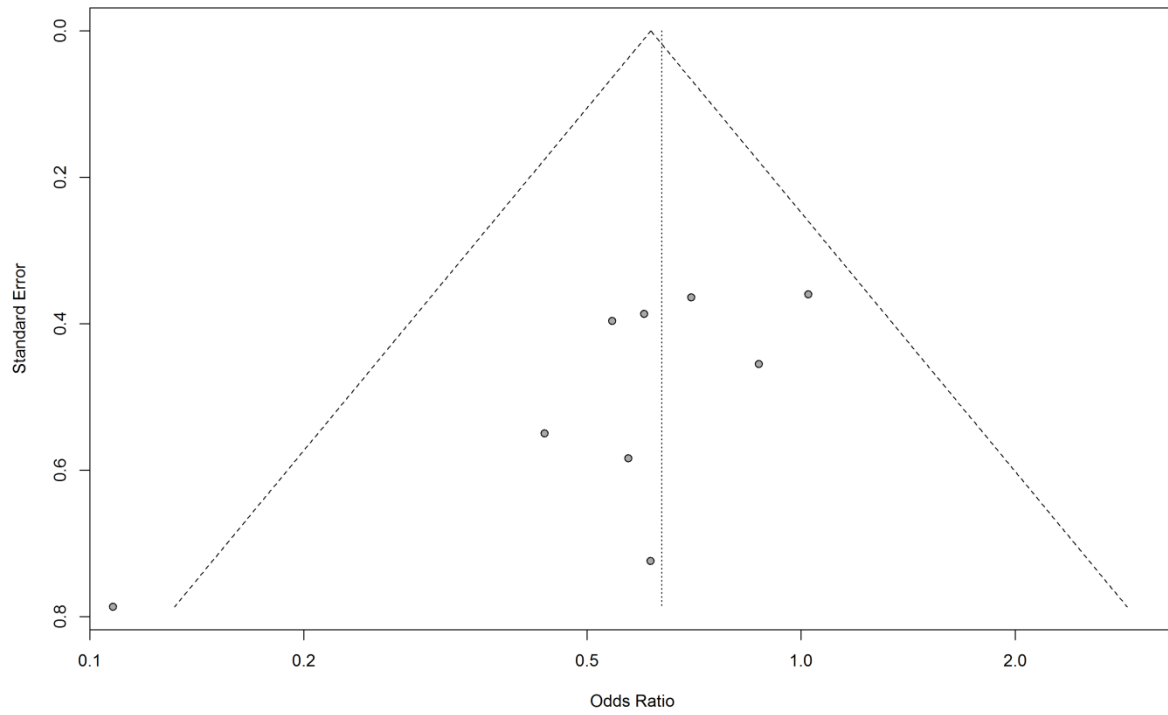

Supplement: S9 Fig — (PDF) [file pone.0231816.s017.pdf]

2.10. Supplementary Figure 10. Funnel plot for publication for house dust mite

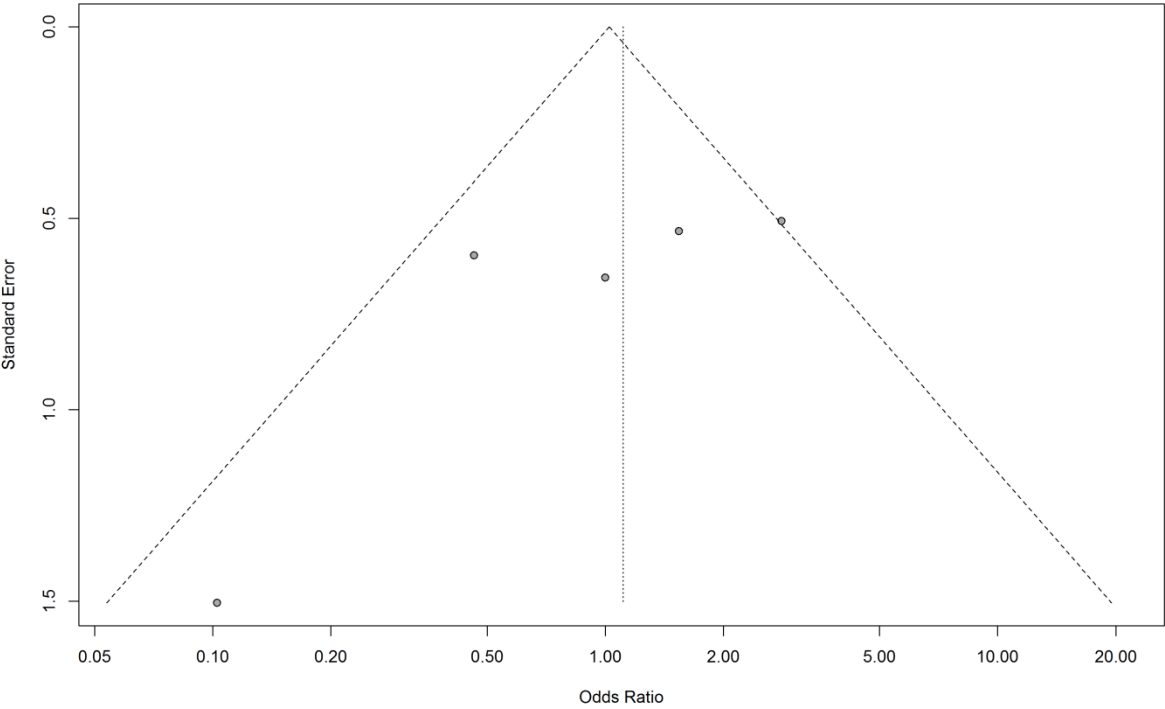

Supplement: S10 Fig — (PDF) [file pone.0231816.s018.pdf]

2.11. Supplementary Figure 11. Funnel plot for publication for positive serum test for food

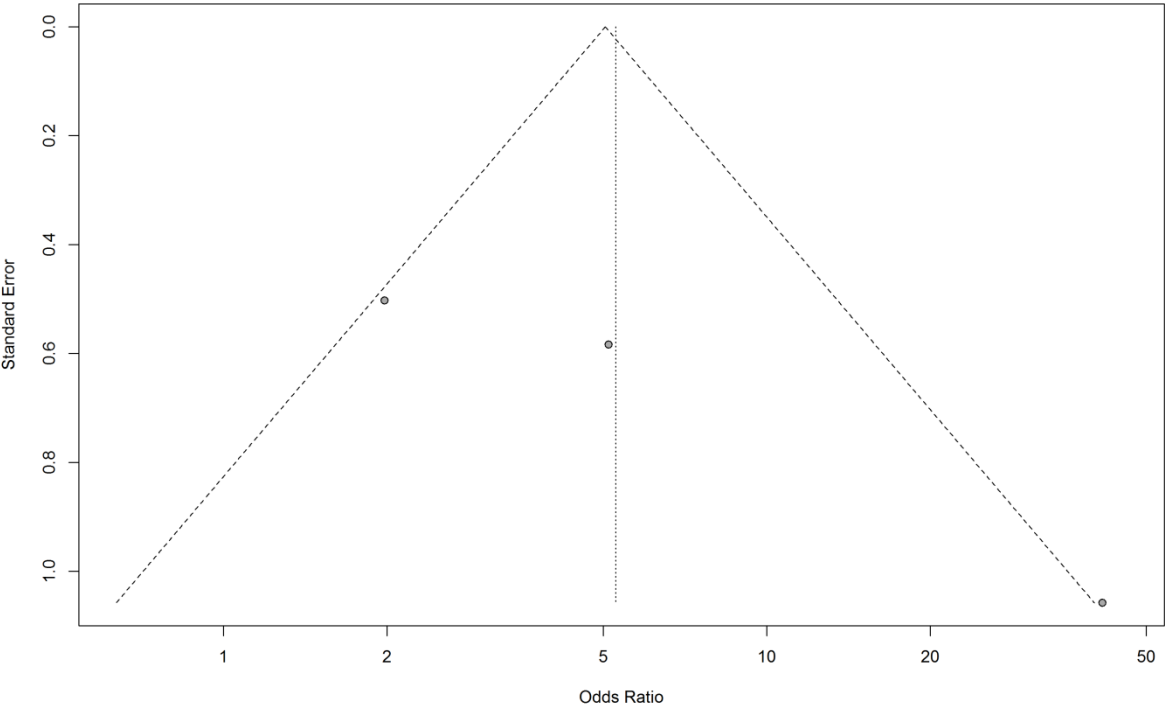

Supplement: S11 Fig — (PDF) [file pone.0231816.s019.pdf]

2.12. Supplementary Figure 12. Funnel plot for publication for positive serum test for inhalants

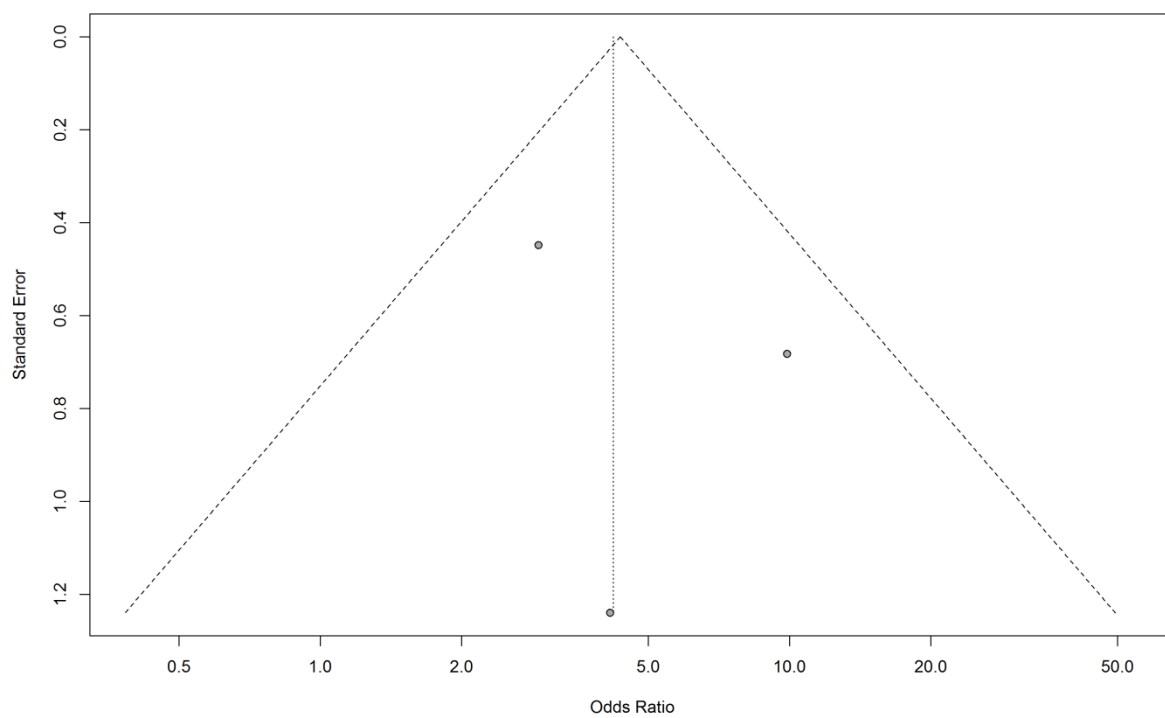

Supplement: S12 Fig — (PDF) [file pone.0231816.s020.pdf]
