# Supplementary material for: Association of early viral lower respiratory infections and subsequent development of atopy, a systematic review and meta-analysis of cohort studies
Source: PLoS One. 2020 Apr 24;15(4):e0231816. doi: 10.1371/journal.pone.0231816 (PMC7182231; doi:10.1371/journal.pone.0231816)
Supplement: Attachment — Submitted filename: Response to Reviewers PONE-D-19-27663.docx [file pone.0231816.s022.docx]

Reviewer #1: (No Response)

We thank the reviewers for their relevant comments.

Reviewer #2: The manuscript is much improved after the revision. The authors have addressed all my questions. I have no further comments.

We thank the reviewers for their relevant comments.

Reviewer #3: The most relevant problem there is the level of evidence. That is the measure of association do not derive from multivariate analysis of original studies but from multivariate metaregression which is totally different. This should be' clearly stated.

We thank to the reviewer for this suggestion. We had previously specified in the abstract that we had conducted multivariate metaregression (Page 2, Line 30). We have now precise also in Methods section that we have performed multivariate metaregression (Page 8, Line 157).
